# Familial Patterns of Oral Microbiota: A Comparison among Children with S-ECC, their Parents, and Parents of Caries-free Children

**DOI:** 10.1007/s00284-026-05212-6

**Published:** 2026-11-03

**Authors:** Yao Wu, Sifan Wang, Chang-hai Lyu, Hai Ming Wong, Ni Zhou

**Affiliations:** 1 Department of Pediatric Dentistry, Hospital of Stomatology, Zhongshan City, China; 2 Department of Pediatric Dentistry, School and Hospital of Stomatology, Yunnan Key Laboratory of Stomatology Yunnan, Kunming Medical University, Kunming, China; 3 Division of Paediatric Dentistry & Orthodontics, Faculty of Dentistry, Dental Hospital, The University of Hong Kong, 2/F Prince Philip, Pokfulam, Hong Kong SAR China

## Abstract

Severe early childhood caries (S-ECC), a prevalent dental disease affecting preschool children, can be influenced by multiple factors, including oral microbiota. Although maternal–child oral microbial similarity has been investigated, few studies have directly compared the microbiota of S-ECC families with that of parents caring for caries-free children. In this study, dental caries status and salivary pH were compared among children diagnosed with S-ECC (*n* = 15), their fathers (*n* = 15), mothers (*n* = 15), and fathers (*n* = 14) and mothers (*n* = 15) of caries-free children using linear mixed-effects models. No statistical differences in microbial diversity were observed among children, their fathers, and mothers, whereas parents of caries-free children exhibited higher Shannon diversity than S-ECC families. S-ECC families also showed higher abundances of *Leptotrichia* and *Streptococcus mutans* than parents of caries-free children (*p* < 0.05). Additionally, parents of S-ECC children showed higher relative abundance of *Fusobacterium* than mothers of caries-free children (*p* < 0.05). Significant crude correlations were observed in the relative abundance of (i) *Veillonella* among child-mother pairs (*r* = 0.62, *p* < 0.05), (ii) *Haemophilus* among both child-mother pairs (*r* = 0.52, *p* < 0.05) and child-father pairs (*r* = 0.65, *p* < 0.01), and (iii) *Actinomyces* among mother-father pairs (*r* = 0.63, *p* < 0.05). However, these associations did not remain statistically significant after FDR correction for multiple comparisons. The principal findings indicated that family units showed similarities in several oral microbial outcomes, supporting further exploration of family-centered interventions for caries management. Prospective studies with large sample sizes are warranted to determine the intra-family correlations of oral microbial profiles.

## Introduction

Dental caries is one of the most prevalent chronic diseases affecting individuals across all age groups. A cross-sectional study comparing dental caries status among children at various dental stages revealed that children with primary dentition were more likely to have dental caries than those with permanent dentition (OR = 7.62, 95% CI 5.22–11.11) [1]. The term, early childhood caries (ECC) is commonly used to describe caries in primary teeth among children under the age of 6 [2]. Severe early childhood caries (S-ECC) presents with more severe clinical manifestations than ECC, typically requiring comprehensive dental management. The prevalence of S-ECC was reported to be 41.2% [3]. A higher occurrence of ECC is commonly observed among children with irregular dental attendance, inappropriate dietary habits, bottle-feeding habits, poor oral hygiene practices, genetic developmental defects affecting the enamel, or those from socioeconomically disadvantaged groups [4–6]. ECC negatively impacts children’s physiological development by impairing masticatory function and speech development, while also causing psychological distress, reducing oral health-related quality of life (OHRQoL), and may require dental rehabilitation under general anesthesia [7, 8].

The oral cavity serves as a complex habitat where microorganisms interact with the host, and if the equilibrium is disrupted, this can lead to the onset or progression of oral diseases [9]. The onset of S-ECC has also been demonstrated to correlate with aberrant alterations in the oral microbial community of children [10, 11]. It has been reported that early childhood infections and high levels of *Streptococcus mutans* colonization are significantly correlated with the prevalence and severity of ECC [12]. While certain studies have reported the potential involvement of other bacteria, such as *Actinomyces* and *Streptococcus sobrinus*, in the initiation and progression of ECC [13, 14]. Additionally, children’s oral microbial communities may be influenced by their parents’ oral microbiota. Jo and colleagues [15] compared oral microbiome profiles among infants, their parents, and unrelated adults, revealing that infants’ oral microbiomes were more similar to their mothers’ than to those of unrelated adults. During the “window of infectivity”, infants were likely to acquire *S. mutans* between 19 and 31 months (median 26 months) [16]. A 24-month longitudinal study revealed dynamic changes in the infant salivary microbiome, showing that children of mothers with caries had greater abundances of *Veillonella* and *Actinomyces* [17]. Moreover, a 20-month longitudinal study infants might obtain *S. sobrinus* and *S. mutans* from mothers; however, during follow-ups, some maternal genotypes were lost, and new strains were acquired [18].

Compared with infants, preschool children exhibit distinct characteristics in their dietary habits and daily routines. Unlike infants, who are primarily dependent on their parents or caregivers for feeding and daily care, preschool children experience a significant diversification in their diet and self-care routines, contributing to their growth and development. These changes can contribute to alterations in the oral microbiota. A case-control study [19] reported that *Veillonella*, which had a higher relative abundance among preschoolers without caries, showed a negative correlation with sugary snacking frequency, whereas *Fusobacterium* and *Neisseria*, which had higher abundance among preschool children with rampant caries, showed a positive correlation with sugary snacking frequency. Variations in oral microbiota between children with and without dental caries have been extensively studied in prior research [13, 19–21]. The oral microbiota of infants and parents have also been well documented [15–18]. However, few studies have compared microbial differences among parents caring for preschool children with and without dental caries.

To address this gap, this study investigated the oral microbial communities of preschool children with S-ECC and compared their microbial profiles with those of their biological parents and the parents of caries-free children. We hypothesized that oral microbial profiles in children with S-ECC would not differ statistically from those of their parents, while greater microbial diversity would be observed between S-ECC children and the parents of caries-free children. Furthermore, a recent case-control study [22] reported that primary caregivers of children with ECC had higher odds of caries than those caring for caries-free children, suggesting that children’s caries status may be more closely associated with their primary caregivers’ than with other adults’. Since caries can be initiated by cariogenic bacteria, we anticipated that a higher abundance of cariogenic species, such as *S. mutans*, would be detected among parents of children with S-ECC compared with parents of caries-free children.

## Materials and Methods

### Study Design and Participant Recruitment

The participants of this cross-sectional study included preschool children diagnosed with S-ECC, their parents, and parents of caries-free children. Preschoolers with S-ECC were screened in the Department of Pediatric Dentistry, Affiliated Stomatological Hospital of Kunming Medical University, Yunnan, China. After screening, an information sheet was provided to their parents, outlining the study’s main purpose and procedures. For children with S-ECC, both mothers and fathers were invited to participate in this study at the hospital. Meanwhile, parents of caries-free children were recruited from local kindergartens. The information sheet was delivered to parents via the kindergartens.

S-ECC was diagnosed in accordance with the following criteria: “i) any sign of smooth-surface caries in a child younger than three years of age, ii) from ages three through five, one or more cavitated, missing (due to caries), or filled smooth surfaces in primary maxillary anterior teeth, or iii) a decayed, missing, or filled score of greater than or equal to four (age three), greater than or equal to five (age four), or greater than or equal to six (age five)” [23]. The inclusion criteria for children with S-ECC were i) aged between 2 and 5 years; ii) diagnosed with S-ECC by trained pediatric dentists; iii) living with parents; iv) no history of previous dental treatment. The exclusion criteria for children and adults were i) having received antibiotics within the past 3 months; ii) undergoing orthodontic treatment; iii) having long-term systemic diseases or developmental disabilities; iv) children not residing with biological parents; and v) parents with limited ability to provide informed consent.

This study was reviewed and approved by the local Ethics Committee of the Affiliated Stomatological Hospital of Kunming Medical University (KYKQ2021MEC089). It was conducted and reported in accordance with the Strengthening the Reporting of Observational Studies in Epidemiology (STROBE) guidelines [24]. As the children involved were aged 2–5 years, written informed consent was obtained from all the parents prior to the study’s commencement. During the dental examination and plaque sample collection, we used tell–show–do (TSD) behavior management techniques and explained the study’s purpose and procedures to the children in an age-appropriate manner. If a child was uncooperative or unwilling to participate, he or she was excluded from the study, even if parental consent had been obtained.

### Sample Size and Statistical Power

The primary outcome was the Shannon index. Oral microbial profiles were compared across 5 groups: children with S-ECC (CC group), fathers of children with SECC (CF group), mothers of children with SECC (CM group), fathers of caries-free children (FF group), and mothers of caries-free children (FM group). Since few studies have compared oral microbial profiles across these 5 groups, we did not conduct a formal sample size calculation based on existing evidence. However, the post-hoc power analysis was conducted using G*Power version 3.1.9.6. Based on the Shannon index scores of the 5 groups, the partial η² was 0.64 and the effect size was 1.33, yielding overall power > 99% across the 5 groups, indicating the current sample size was sufficient to detect an overall difference across the five groups.

### Dental Examination

Two calibrated pediatric dentists who participated in a training session on oral health examinations conducted intra-oral examinations using probes and disposable mirrors. Caries status was assessed in accordance with the World Health Organization (WHO) criteria [25]. The decayed, missing due to caries, and filled (dmft) index was calculated for preschool children, while the decayed, missing due to caries, and filled (DMFT) index was used for parents. Children with dmft = 0 were regarded as “caries-free”. 10% of the participants were randomly selected to assess intra-examiner reliability, and the kappa value for caries assessment was over 0.91. Additionally, the participants were instructed to relax for five minutes and maintain an upright sitting position in the chair. Unstimulated saliva was collected, and the salivary pH was immediately measured using the protocol described in our previous publication [19].

### Supragingival Plaque Collection and 16 S rRNA Sequencing

Immediately after dental examination, supragingival dental plaque was collected from both the buccal and lingual surfaces of all deciduous teeth in child participants, following the protocol described in a prior publication [19]. For adults, dental plaque was collected from the index tooth surfaces used to assess the Simplified Oral Hygiene Index (OHI-S) [26], including the buccal surfaces of teeth 16, 11, 26, and 31 and the lingual surfaces of teeth 36 and 46, in line with the method described in a prior study [27]. Saliva and plaque samples were collected on the same day around 10:00–11:00 am or 14:00–16:00 pm under participants’ routine conditions, without altering their normal diet or toothbrushing routines. The obtained plaque samples were kept in 2.0 mL cryotubes, immediately frozen, and transferred to liquid nitrogen at -196 °C [19]. Microbial DNA was extracted from 74 supragingival plaque samples using the cetyltrimethylammonium bromide (CTAB) method, following the manufacturer’s instructions. The V3-V4 regions of bacterial 16 S rRNA genes were amplified. The polymerase chain reaction (PCR) mixture was amplified using the method described by Lei and colleagues (2022) [22]. The products were purified, and the libraries were sequenced on Illumina NovaSeq 6000 (Illumina Inc., CA, USA). The raw Illumina sequencing data have been submitted to the SRA database (PRJNA1276421).

### Data Analysis

Data entry was cross-checked by two investigators (WY & ZN). The intra-examiner reliability was assessed using the Kappa statistic. Sequencing data processing involved quality control using FASTP [28] (v 0.18.0) to generate clean reads, followed by read assembly with FLASH [29] (v 1.2.11) and stringent quality trimming according to Bokulich et al.’s protocol [30], DADA2 [31] (v1.14.1) was then employed for primer removal, error modeling, and Amplicon Sequence Variant (ASV) generation, with chimera detection performed by UCHIME [32], before taxonomic annotation against the SILVA [33] database (v138.2) using the RDP [34] classifier (v2.2). Alpha diversity was assessed by the Shannon index. Beta diversity was evaluated using Principal Coordinates Analysis (PCoA) based on weighted UniFrac distances and Permutational Multivariate Analysis of Variance (PERMANOVA) using the Vegan package [35]. Microbial composition was visualized for the top 10 genera using Krona [36] (v2.6) and ggplot2 package [37]. Comparisons of caries status, salivary pH levels, Shannon diversity index, and the relative abundances of selected taxa were performed using IBM SPSS Statistics 28.0 (IBM Corp., Armonk, NY, USA) and R software (version 4.5.3). Normality was analyzed using Shapiro-Wilk tests. Differences in DMFT/dmft, salivary pH, Shannon diversity, and the relative abundances of the top 10 genera and *S. mutans* among the five groups were assessed using linear mixed-effects models (LMMs). Since children and their parents from the same family were not statistically independent, family ID was included as a random effect to account for within-family correlation. Bacterial relative abundance data were log10-transformed before modeling. Study group was included as a fixed effect, and pairwise comparisons were performed using estimated marginal means when overall group differences were significant. To account for multiple testing across bacterial taxa, *p*-values were adjusted using Benjamini-Hochberg false discovery rate (FDR) correction. Associations in the relative abundances of the top 10 bacterial genera among children, fathers, and mothers within the same family were assessed using Spearman’s rank correlation analysis. To account for multiple testing across taxa and family-member pairs, p-values were adjusted using the FDR procedure. All statistical tests were two-tailed. The significance level was set at 0.05.

## Results

### Characteristics of the Participants

A total of 74 participants were recruited, including children with S-ECC (CC group, *n* = 15), fathers of children with SECC (CF group, *n* = 15), mothers of children with SECC (CM group, *n* = 15), fathers of caries-free children (FF group, *n* = 14), and mothers of caries-free children (FM group, *n* = 15). Among them, 48.6% (*n* = 36) were males, and the recruited children had an average age of 3.07 ± 0.88 years and a dmft score of 11.53 ± 4.47. There were no significant differences in the ages of parents whose children were diagnosed with or without caries. The average DMFT score was slightly higher among parents of children with S-ECC than among parents of caries-free children; however, this difference was not statistically significant. A significantly higher salivary pH was observed among fathers of caries-free children compared with mothers of children with S-ECC (7.28 ± 0.44 vs. 6.89 ± 0.28, adjusted *p* = 0.022; Table 1).

**Table 1:** Age and oral conditions among the children and parents (*n* = 74)

	CC (*n* = 15)	CF(*n* = 15)	CM (*n* = 15)	FF (*n* = 14)	FM (*n* = 15)
Age	3.07 ± 0.88	36.80 ± 7.53	34.00 ± 6.31	35.07 ± 3.81	33.93 ± 4.11
dmft/DMFT	11.53 ± 4.47	2.07 ± 2.43	3.93 ± 2.28	1.79 ± 2.04	3.73 ± 3.03
Salivary pH	6.95 ± 0.49	6.82 ± 0.28	6.89 ± 0.28*	7.28 ± 0.44*	6.92 ± 0.40

Data were presented as mean ± SD. CC-children with caries, CF-fathers of children with caries, CM-mothers of children with caries; FF-fathers of caries-free children, FM- mothers of caries-free children. *Statistical differences were detected in salivary pH between mothers of children with caries and fathers of caries-free children (multiple comparisons using FDR, with adjusted *p* = 0.022)

### Sequencing Result

**Fig. 1:**
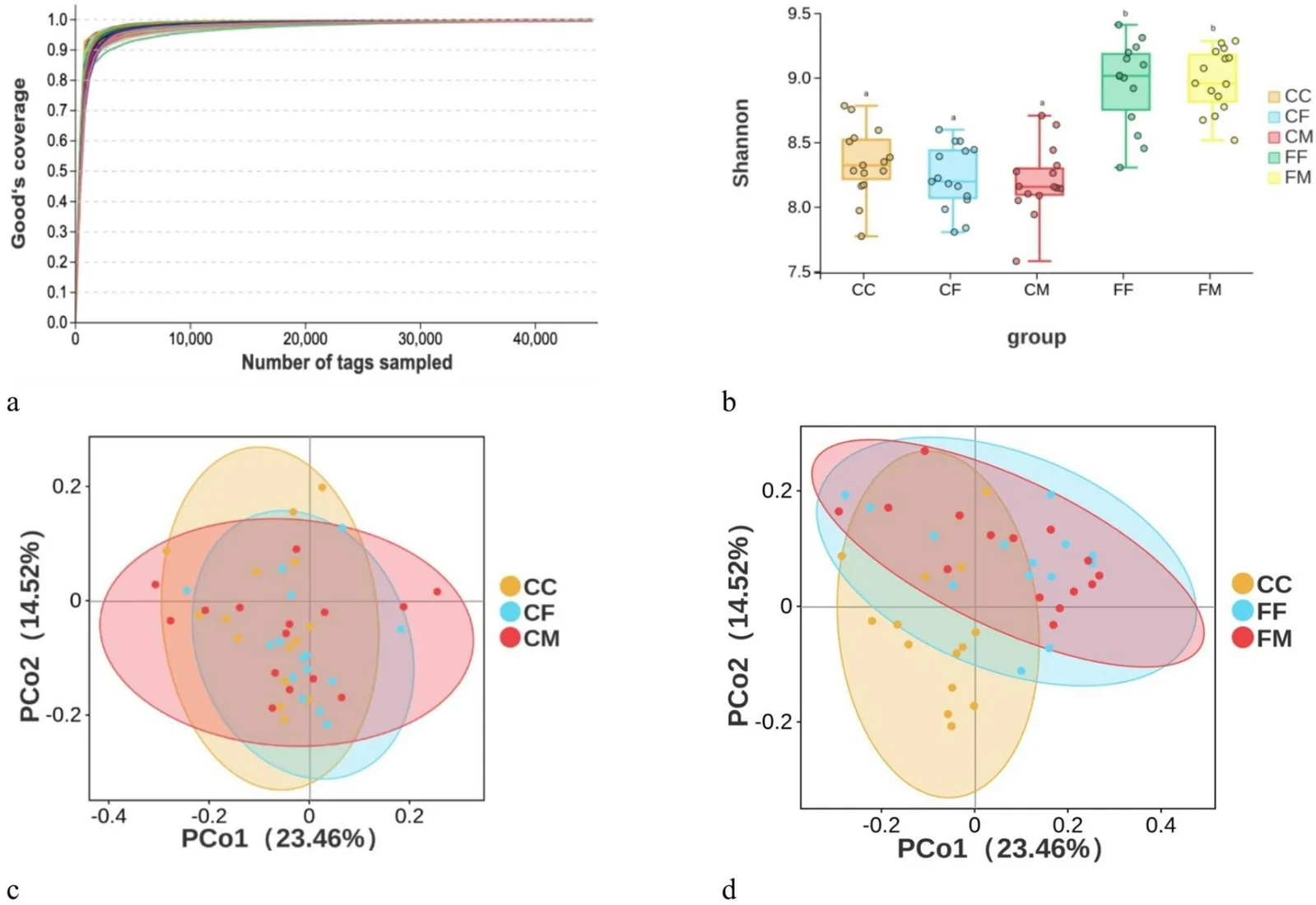
Rarefaction curves and oral microbial diversities among children and parents. (**a**)Rarefaction curves, demonstrating stable plateau achieved among all the dental plaque samples; (**b**) Comparison of alpha-diversity, showing no statistical differences in Shannon index between children and their own parents, while higher Shannon index were observed among parents of caries-free children when compared to families of children with S-ECC (Linear mixed-effects model (LMM) using FDR correction, *p* < 0.001); (**c**) No statistically significant differences were observed in beta-diversity between children with S-ECC and their own parents using PCoA on weighted UniFrac distances (PERMANOVA test, R^2^ = 0.0605, *p* > 0.05); (d) Significant differences in beta-diversity between children with S-ECC and parents of caries-free children (PERMANOVA test, R^2^ = 0.1207, *p* = 0.001). Note: CC-children with S-ECC, CF-fathers of children with S-ECC, CM-mothers of children with S-ECC; FF-fathers of caries-free children, FM- mothers of caries-free children

A total of 5,762,942 high-quality sequence tags were generated from 16 S rRNA gene sequencing. The rarefaction curve analysis was employed to evaluate sequencing saturation and indirectly compare species richness across groups. In this study, the number of observed ASVs reached a stable plateau as sequencing depth increased (Fig. 1a), indicating adequate sequencing coverage, with additional sequencing yielding few new species.

### Comparison of Oral Microbial Diversity among Children and Parents

Statistical differences were detected in the Shannon index across the 5 groups (*p* < 0.001). Multiple comparisons showed no statistical differences in the Shannon index between children and their own parents. Likewise, no significant difference was observed in Shannon index between fathers of caries-free children and mothers of caries-free children (*p* > 0.05, Fig. 1b). However, higher Shannon values were observed among parents of caries-free children than families of children with S-ECC. Those findings indicated more species or a more even distribution among parents of caries-free children compared with S-ECC children and their parents.

The PCoA results showed that Principal Component 1 (PCo1) and PCo2 accounted for 23.46% and 14.52% of the total variation, respectively. No statistically significant differences were observed between S-ECC children and their own parents (*p* > 0.05, Fig. 1c), whereas significant differences were detected between children with S-ECC and parents of caries-free children (*p* = 0.001, Fig. 1d). In Fig. 1d, S-ECC children clustered in the negative half-axis of the vertical coordinate, while parents of caries-free children clustered in the positive half-axis of the horizontal coordinate.

### Oral microbiome among Children with S-ECC, their Parents, and Parents of Caries-free Children

**Fig. 2:**
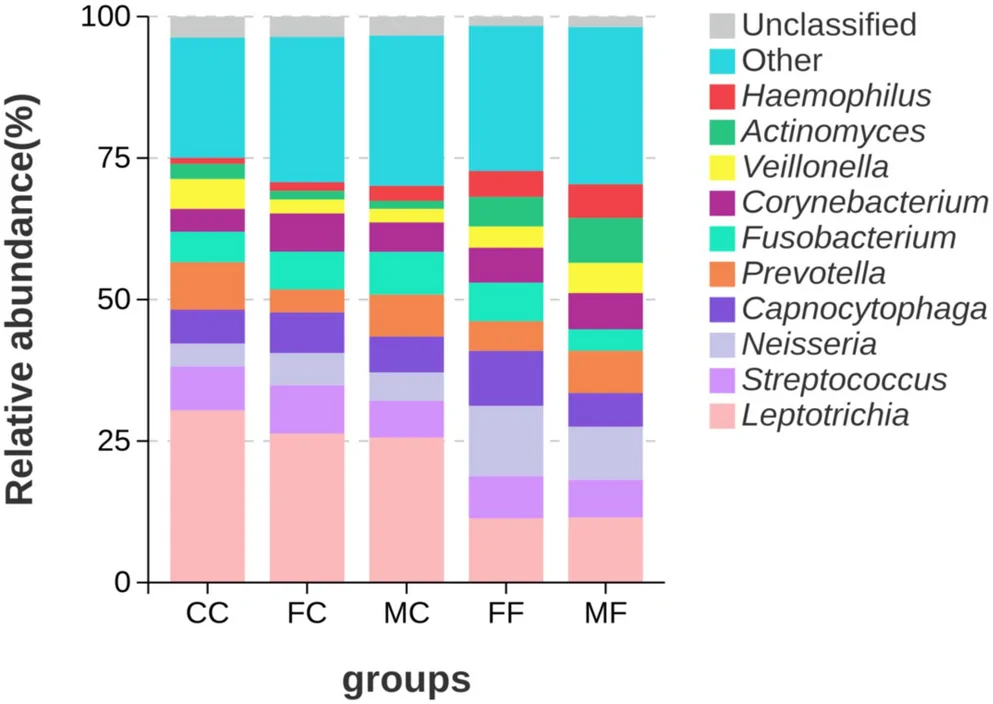
Top 10 genera relative abundance among the participants. Higher relative abundance of *Leptotrichia* was observed in CC, CF, and CM groups, while *Actinomyces* and *Haemophilus* were more enriched among FF and FM groups. CC-children with S-ECC, CF-fathers of children with S-ECC, CM-mothers of children with S-ECC; FF-fathers of caries-free children, FM-mothers of caries-free children

The community composition analysis revealed that the Top 10 most abundant genera among children and parents were *Leptotrichia*,* Streptococcus*, *Neisseria*, *Actinomyces*, *Haemophilus*, *Prevotella*, *Fusobacterium*, and *Veillonella* (Fig. 2). Among these, we observed significant differences in two genera across the five groups: *Leptotrichia* (*p  = 0.026*) and *Fusobacterium* (*p* = 0.043).

*Leptotrichia*, the most abundant genus, exhibited higher relative abundances in ECC families (CC, CF, CM) than in parents of caries-free children (*p  = 0.026*). Furthermore, pairwise comparisons showed that the relative abundance of *Fusobacterium* was significantly higher among mothers and fathers of S-ECC children than among mothers of caries-free children (*p* < 0.05, Table 2). Conversely, the bar plot showed higher relative abundances of *Neisseria*, *Actinomyces*, and *Haemophilus* in parents of caries-free children than in S-ECC families; however, the differences were not statistically significant (*p* > 0.05).

**Table 2:** Inter-group comparisons of oral microbial composition at the genus level (*n* = 74)

Genus	Relative abundance (%)					*p*-value (FDR)	Multiple comparisons
	CC	CF	CM	FF	FM		
*Leptotrichia*	30.40 ± 13.78	26.31 ± 11.36	25.60 ± 11.23	11.32 ± 9.19	11.48 ± 8.45	0.026	CC, CF, CM > FF, FM
*Streptococcus*	7.78 ± 6.06	8.53 ± 5.25	6.50 ± 5.77	7.48 ± 6.12	6.65 ± 5.19	0.652	/
*Neisseria*	4.04 ± 3.30	5.71 ± 2.78	5.02 ± 3.24	12.41 ± 10.74	9.40 ± 11.06	0.394	/
*Prevotella*	8.41 ± 7.06	4.08 ± 3.52	7.43 ± 10.10	5.25 ± 9.02	7.50 ± 8.83	0.268	/
*Fusobacterium*	5.39 ± 3.68	6.67 ± 2.27	7.53 ± 2.96	6.80 ± 7.85	3.77 ± 3.49	0.043	CF, CM > FM
*Veillonella*	5.28 ± 5.46	2.46 ± 1.86	2.39 ± 1.99	3.75 ± 5.49	5.33 ± 4.77	0.504	/
*Actinomyces*	2.68 ± 4.80	1.53 ± 1.65	1.42 ± 1.17	5.22 ± 8.90	7.94 ± 10.84	0.187	/
*Haemophilus*	1.07 ± 1.79	1.54 ± 1.68	2.65 ± 5.02	4.59 ± 5.34	5.96 ± 6.86	0.187	/
*Capnocytophaga*	5.97 ± 4.14	7.16 ± 4.53	6.33 ± 3.23	9.71 ± 8.14	5.95 ± 4.12	0.652	/
*Corynebacterium*	4.02 ± 3.18	6.75 ± 6.71	5.22 ± 3.39	6.19 ± 5.35	6.40 ± 4.18	0.652	/

Data were presented as mean ± SD. CC-children with caries, CF-fathers of children with caries, CM-mothers of children with caries; FF-fathers of caries-free children, FM- mothers of caries-free children

**Fig. 3:**
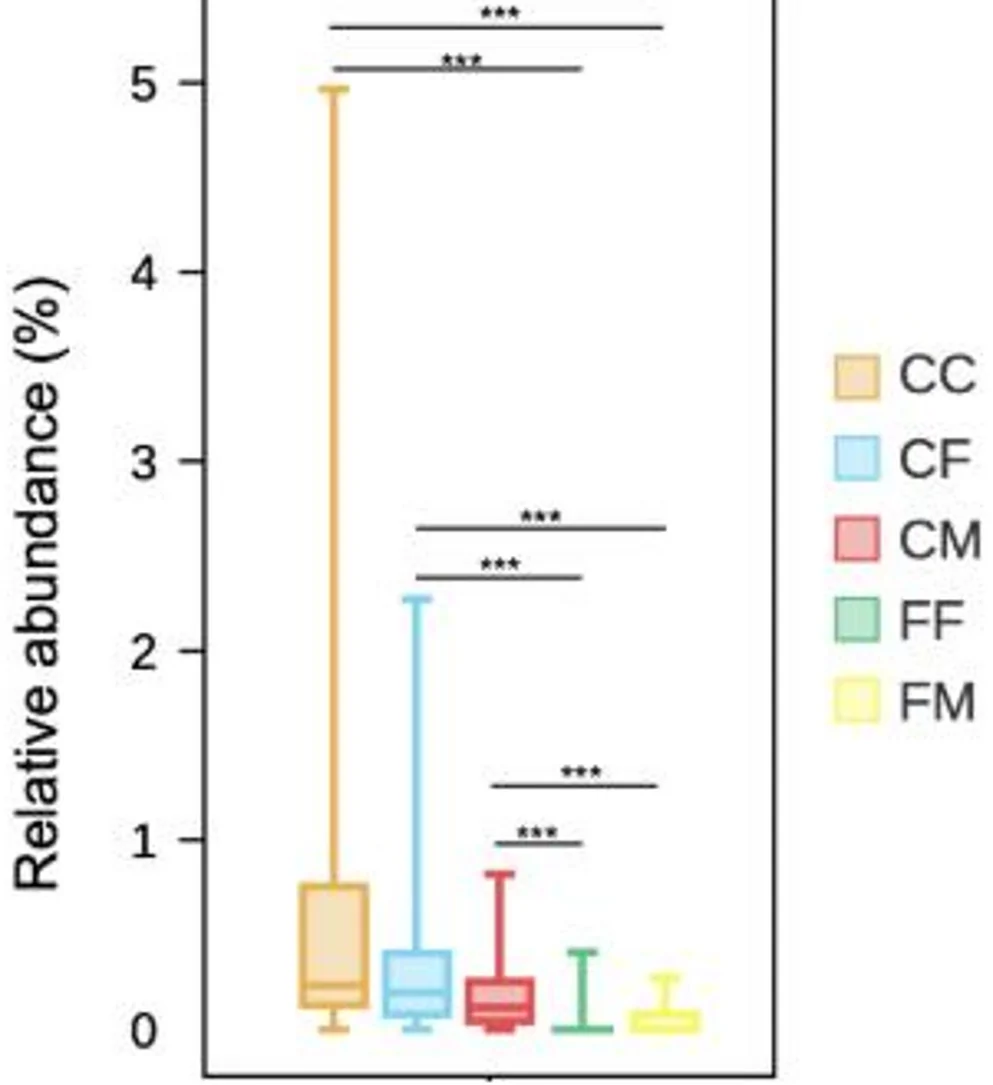
Comparison of the relative abundance of *S. mutans* among participants. The multiple comparisons (FDR corrections) showed that the CC group had a higher relative abundance of *S. mutans* than FF (*p* < 0.001) and FM (*p* < 0.001) groups; the CF group also had a higher relative abundance of *S. mutans* than FF (*p* < 0.001) and FM (*p* < 0.001) groups; the CM group had a higher relative abundance of *S. mutans* than the FF group (*p* < 0.001) and FM (*p* < 0.001) groups. CC-children with caries, CF-fathers of children with caries, CM-mothers of children with caries; FF-fathers of caries-free children, FM- mothers of caries-free children. Group differences were evaluated using a linear mixed-effects model (LMM), followed by FDR-adjusted pairwise comparisons. *p < 0.05, ** *p* < 0.01, *** *p* < 0.001

Given that *S. mutans* has been well documented as a cariogenic bacterium, its distribution was also compared among participants. Significant differences in the relative abundance of *S. mutans* were detected across the five groups (*p* < 0.001). Pairwise comparisons showed that the relative abundance of *S. mutans* was significantly higher in children with S-ECC (CC group), their fathers (CF), and their mothers (CM) than in fathers and mothers of caries-free children (all adjusted *p* < 0.001, Fig. 3). However, no significant differences were detected in the relative abundance of *S. mutans* between S-ECC children and their parents (*p* > 0.05). Additionally, a similar relative abundance of *S. mutans* was observed in couples whose children were affected with S-ECC. Likewise, no significant differences were detected in the relative abundance of *S. mutans* between fathers and mothers whose children were caries-free (*p* > 0.05; Fig. 3).

### Intra-family Correlations of Oral Microbial Profiles

**Fig. 4:**
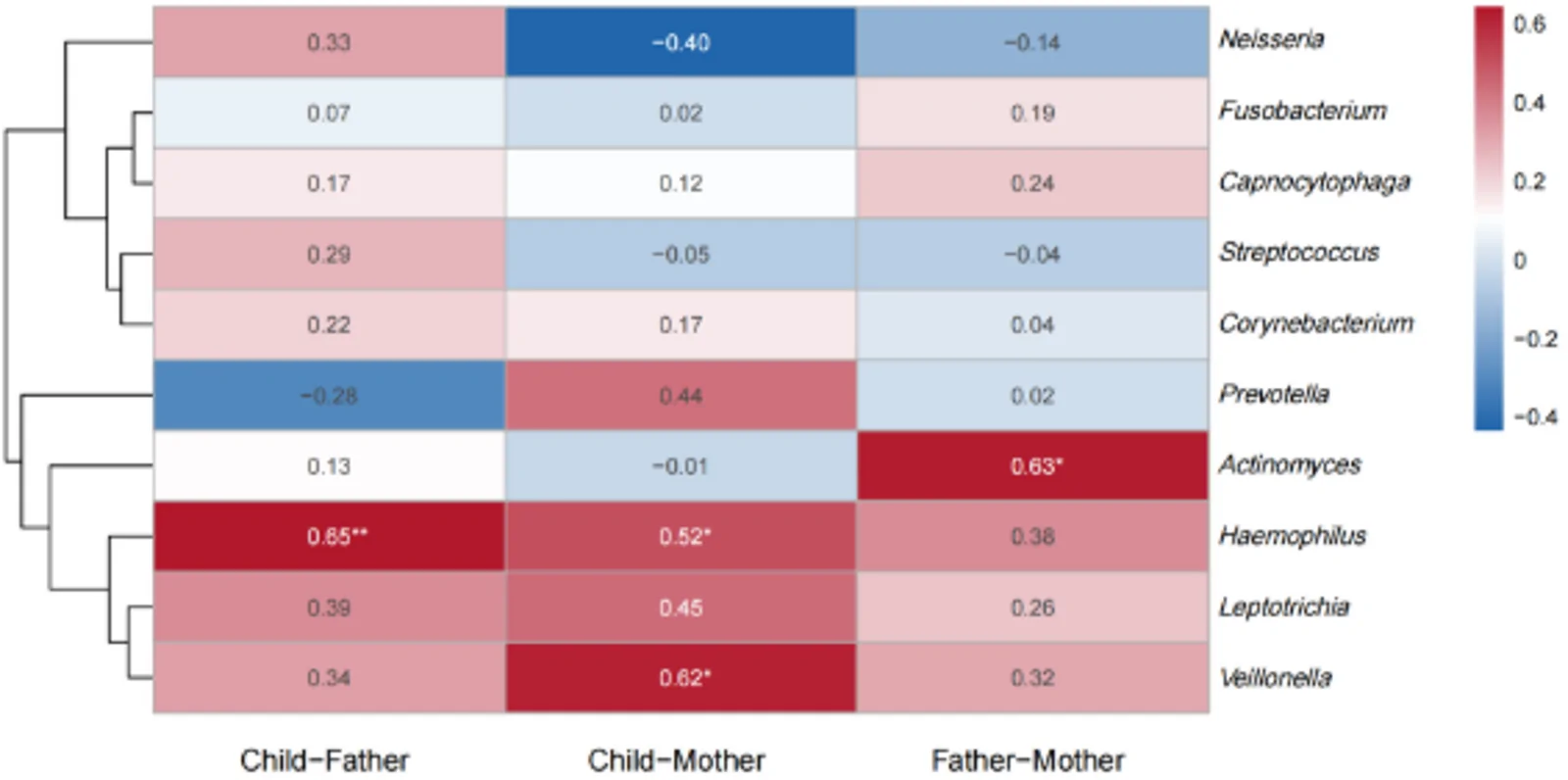
Spearman’s rank correlations in relative abundance of the Top 10 genera between children, mothers, and fathers (*n* = 15 families) *crude p-value < 0.05, ** crude p-value < 0.01. None of the observed associations remained statistically significant after FDR correction (all adjusted p-values > 0.05)

Figure 4 shows the Spearman correlations of the relative abundances of the Top 10 most abundant genera among children, fathers, and mothers in 15 families. Among these, a significant crude correlation (*r* = 0.62, *p* < 0.05) was observed in the relative abundance of *Veillonella* between children and their mothers, whereas this relationship was non-significant between children and fathers, nor between mothers and fathers. Significant correlations were found in the relative abundance of *Haemophilus* among both child-mother pairs (*r* = 0.52, *p* < 0.05) and child-father pairs (*r* = 0.65, *p* < 0.01). Additionally, *Actinomyces* showed a significant correlation within the couples (*r* = 0.63, *p* < 0.05). However, these associations did not remain statistically significant after FDR correction for multiple comparisons (all FDR-adjusted p-values > 0.05).

## Discussion

Evidence suggests that multiple parent-related factors can affect children’s caries status. A population-based study demonstrated that mothers’ untreated dental caries was associated with children’s caries status [38]. Genetically, children may inherit predispositions to certain dental conditions from their parents. Familial aggregation studies identified several caries markers, indicating that 13q31.1 was potentially associated with caries susceptibility, while 2.5 bp and 2 bp band expressions might be related to low dental caries susceptibility [39–41]. Additionally, the colonization of cariogenic bacteria, for instance, *S. mutans*, *Lactobacillus casei*, and *L. acidophilus*, was documented to be associated with the host HLA class II and TNFα genetic profiles [42]. To the best of our knowledge, few studies have compared oral microbiota disparities among S-ECC children, their fathers, their mothers, and parents of preschool children without caries.

In this study, the familial analysis based on triads (father, mother, and child) revealed no significant differences in alpha or beta diversity among S-ECC children, their mothers, and their fathers (*p* > 0.05). Likewise, a recent study by Rothman and colleagues [43] found no significant differences in oral microbial alpha diversity between children and caregivers (unspecified as mothers or fathers), consistent with our findings. However, they identified statistical differences in beta diversity between children and caregivers (R^2^ = 0.02, F = 10.56, *p* < 0.001) [43]. Additionally, a study [15] involving 18-month-old infants demonstrated significantly lower microbial diversity in infants compared with their fathers and mothers. Another study [44] compared the oral microbiota among 4-month-old and 18-month-old infants and their mothers, showing a significantly lower Shannon index in infants than in mothers; however, the Shannon index increased significantly at 18 months, and the microbial communities at 18 months overlapped more with the maternal microbiome than at 4 months. Those findings indicated longitudinal changes in the oral microbiota of infants in early childhood.

Although no significant differences in oral microbial diversity were observed between children with S-ECC and their own parents, inter-family comparisons revealed that both alpha and beta diversities differed significantly between children with S-ECC and parents of caries-free children. Likewise, a prior study [15] demonstrated that the oral microbial composition was more diverse in children than in unrelated adults, compared with their own mothers, indicating that children’s oral microbiota might be influenced by parental microbial communities. Furthermore, in our study, parents of children with S-ECC exhibited lower alpha diversity indices compared to parents of caries-free children. Typically, individuals with dental caries have less diverse oral microbiota than those in a healthy state [9]. These findings indicate that familial and caries-status-associated factors contribute to variations in oral microbial diversity.

In the present study, we observed a similar distribution pattern in the relative abundance of *Leptotrichia* and *S. mutans*. We detected no significant intra-family variation in the relative abundance of *Leptotrichia* or *S. mutans*, as children with S-ECC showed levels comparable to those of both their mothers and fathers. However, both *Leptotrichia* and *S. mutans* were enriched in S-ECC families compared with parents of caries-free children (Table 2; Fig. 3). This enrichment in S-ECC families may reflect oral health status within the target families. A recent case-control study showed that caregivers who cared for preschoolers with ECC had higher caries prevalence and higher DMFT scores than caregivers of ECC-free children [22]. A cross-sectional study showed positive correlations in dental caries status among child-father dyads (r_s_=0.30, 95%CI:0.10–0.47) and child-mother dyads [27] (r_s_=0.29, 95% CI:0.16–0.41). *S. mutans* is reported to be associated with the prevalence and severity of dental caries [12]. In our study, *Leptotrichia* was the most abundant genus in S-ECC families. Similarly, another study among Chinese children [45] identified *Leptotrichia* as the most abundant genus in children with S-ECC and suggested its potential as a biomarker for caries in preschool-aged children. Since both *S. mutans* and *Leptotrichia* were frequently detected in individuals with caries, parents of children with caries were more likely to have caries than parents of caries-free children. This may partially explain the enrichment of these bacteria in S-ECC families. Additionally, several studies reported that *S. mutans* can be vertically transmitted from mothers during infancy [16, 18]. Transmission of *S. mutans* between spouses was also documented [46]. These findings indicated that *Leptotrichia* and *S. mutans* might be shared within families and could play an important role in the familial aggregation of caries risk, highlighting the importance of considering family-wide microbial profiles in the management of S-ECC.

Interestingly, the caries status of children with S-ECC was significantly more severe (dmft = 11.53) than that of their fathers (DMFT = 2.07) as well as their mothers (DMFT = 3.93). However, no statistically significant differences were observed in salivary pH values, alpha diversity, beta diversity, or the relative abundance of *S. mutans* between children and their parents. It is reported that caries development is closely related to low salivary pH and the presence and abundance of cariogenic bacteria [19, 47, 48]. The significantly higher caries experience in children might be attributed to several factors. Firstly, the children recruited in this study were diagnosed with S-ECC. A previous study [18] reported that almost 30 genera were significantly different between preschoolers with and without dental caries. For instance, *Veillonella* and *Leptotrichia* were more abundant among children without dental caries, whereas *Neisseria* and *Fusobacterium* were enriched among caries-free children. However, this study did not analyze the oral microbiota of caries-free children. Only children with S-ECC were recruited, yielding a high dmft score of 11.53 ± 4.47. If caries-free children had been recruited, different oral microbial profiles might be observed. Secondly, the recruited children were in the primary dentition stage, during which their teeth were less mineralized and more susceptible to demineralization and tooth decay than permanent teeth in adults. Additionally, differences in immune response [49, 50] could contribute to variations in caries severity, as age-related affinity maturation likely enhances the production of more effective antibodies in adults than in young children.

Several limitations existed in this study. First, the sample size was relatively small, with only 15 familial triads (father, mother, and child with S-ECC) recruited. While the rarefaction curve indicated adequate sequencing coverage across the 74 dental plaque samples, the limited sample size limited the feasibility of pairwise comparisons of clinical outcomes across multiple groups. Although the post hoc power analysis yielded an overall power > 99% across the 5 groups, the actual power between S-ECC families and FF/FM groups was only 6% to 32%. These findings suggest that intra-family differences may be relatively small, and the current sample size may be sufficient to detect overall differences across the 5 groups but insufficient to detect intra-family differences. Additionally, caries is a multifactorial disease influenced by dietary habits, oral hygiene practices, fluoride exposure, socioeconomic status, and educational attainment [1, 51]. Although this study found that microbial profiles differed between S-ECC families and parents of caries-free children, the observed differences in microbiota cannot be generalized as a typical family-based pattern, as they may be attributable to confounding factors. There is evidence that smoking, dental treatment, and toothpastes containing inactivated *Lacticaseibacillus paracasei Probio-01* are associated with dynamic changes in the oral microbiota [52–55]. However, we did not screen for whether parents smoked, underwent periodontal treatment, or used special toothpastes. Moreover, we used convenience sampling, and selection bias may have occurred. Future studies should adopt a randomized sampling method and more rigorous eligibility criteria for participant recruitment.

Another limitation was the lack of longitudinal follow-up. Since dental caries is a chronic, multifactorial disease, dynamic changes in oral microbiota and caries progression may occur over time. Given the cross-sectional design, the current findings could not determine whether oral microbial communities were vertically transmitted from parents to children. Moreover, this study used 16 S rRNA sequencing, which is suitable for assessing overall diversity and taxonomic composition of microbial communities among family members. However, Wei et al. [56] incorporated artificial intelligence (AI) techniques into metagenomic and full-length 16 S rRNA gene sequencing, yielding innovative insights into caries etiology. Yang et al. [20] conducted longitudinal analyses of plaque samples from single teeth in 2,504 children with ECC and found significant spatial and temporal heterogeneity in microbial communities across different teeth, tooth positions, and time points within the same child’s oral cavity. In the present study, we mixed dental plaque collected from various teeth and did not detect site-specific variation in supragingival microbial communities. Well-designed longitudinal studies using advanced techniques are recommended in future studies.

## Conclusion

In this study, the oral microbial communities among children with S-ECC, their own parents (both mothers and fathers), and parents of caries-free children were compared. Intra-familial comparisons suggested similar microbial diversity and comparable relative abundances of *S. mutans* and the Top 10 most abundant genera between children with S-ECC and their parents, while significant differences in alpha diversity, beta diversity, and the abundance of *Leptotrichia* and *S. mutans* were observed between children with S-ECC and parents of caries-free children. Notably, parents of children with S-ECC exhibited lower microbial diversity and higher abundances of *Leptotrichia* and *S. mutans* compared to parents of caries-free children. However, given the small sample size of the familial triads, future large-scale, longitudinal studies are essential to confirm these findings. Additionally, the principal findings support further investigation of family-oriented preventive approaches.

## Data Availability

The raw data have been deposited in the SRA database (PRJNA1276421), which can be accessed via https://dataview.ncbi.nlm.nih.gov/object/PRJNA1276421?reviewer=g5f7b8qkrr3s7b7ai412n70trj.
